# Tandem Repeat Diversity in Two Closely Related Hamster Species—The Chinese Hamster (*Cricetulus griseus*) and Striped Hamster (*Cricetulus barabensis*)

**DOI:** 10.3390/biomedicines10040925

**Published:** 2022-04-18

**Authors:** Nadezhda G. Ivanova, Irina V. Kartavtseva, Vera N. Stefanova, Dmitrii I. Ostromyshenskii, Olga I. Podgornaya

**Affiliations:** 1 Laboratory of Noncoding DNA, Institute of Cytology RAS, Saint Petersburg 194064, Russia; vestefan@mail.ru (V.N.S.); necroforus@gmail.com (D.I.O.); opodg@yahoo.com (O.I.P.); 2 Laboratory of Evolutionary Zoology, Federal Scientific Center of the East Asia Terrestrial Biodiversity, Vladivostok 690022, Russia; kartavtseva@biosoil.ru; 3 Department of Cytology and Histology, Faculty of Biology, St. Petersburg State University, Saint Petersburg 199034, Russia

**Keywords:** tandem repeats, satellite DNA, CHO (Chinese hamster ovary cell lines), *Cricetulus griseus*, *Cricetulus barabensis*, FISH

## Abstract

The Chinese hamster (*Cricetulus griseus*) and striped hamster (*Cricetulus barabensis*) are very closely related species with similar karyotypes. The karyotypes differ from each other by one Robertsonian rearrangement and X-chromosome morphology. The level of the tandem repeat (TR) sequences’ evolutional variability is high. The aim of the current work was to trace the TR distribution on the chromosomes of two very closely related species. The striped hamster genome has not yet been sequenced. We classified the Chinese hamster TR in the assemblies available and then compared the mode of the TR distribution in closely related species. Chinese and striped hamsters are separate species due to the relative species specificity of Chinese hamster TR and prominent differences in the TR distribution in both species. The TR variation observed within homologous striped hamster chromosomes is caused by a lack of inbreeding in natural populations. The set of TR tested could be used to examine the CHO lines’ instability that has been observed in heterochromatic regions.

## 1. Introduction

The development of genome sequencing and assembly methods, as well as the progress of methods for processing high-throughput sequencing data, allow the identification and annotation of large tandem repeats (TR), also called satellite DNA (satDNA), in the genome-assembled contigs of different organisms. Specific features of the TR structural organization complicate the assembly, annotation, and mapping of heterochromatic chromosome regions that contain TR as the main component [1]. The bioinformatics approach, which provides TR identification in the genome assemblies, was developed earlier [2]. The term ‘large tandem repeats’, which is used in the present study, can be formalized, since all TR characteristics have a numerical expression. Historically, for some TR sequences the term satellite DNA (satDNA) is used, for example, for hamster HC2sat and Sat5CH in Repbase. The bulk of the information that has been obtained about classical satDNA by the scientific community is applicable to the TR.

It has been known for decades that the centromeres (CEN) of different species from fission yeast to humans contain TR, with TR-enriched pericentromeric (periCEN) regions appearing to be critically important for establishing heterochromatin and proper chromosome segregation [3]. In recent years, TR have taken on new importance due to the understanding that their structure may confer potentially unique functional characteristics [4].

TR DNA is organized as multiple copies of related DNA sequences of a certain size (repeat unit or monomer), arranged in a head-to-tail pattern to form tandem arrays (fields). The TR cloned in the pre-genomic era as satDNA allow us to ascertain that in mammals the main TR (~10%) is located in the periCEN, whereas the CENP-box-containing TR (~1%) is localized in the narrow CEN [1,5]. Bioinformatics methods for the CEN and periCEN TRs’ discrimination have not been developed yet. It is computer-based research that establishes the sequence localization at the CEN-periCEN region, rather than functional checking [6,7,8]. The experimentally cloned CEN TR, which actually work as CEN, are known for several species (mouse, human) but are not yet determined for most other species, i.e., the sequences did not go through functional checking. When satDNA or TR were mapped to the metaphase chromosomes, their position was described as broadly centromeric, i.e., in the primary constriction region [5,9].

Two TR were cloned from the Chinese hamster (*Cricetulus griseus*) genome and placed in Repbase. HC2sat was described as a TR with a 2.8 kb monomer that is mapped to the CEN of chromosome 2. Such a monomer is one of the longest known among mammalian TR. The repetitive elements (ATTT)_n_, (AATG)_n_, and (CA)_n_ are recognized inside this long monomer [10]. The other TR, SatCH5 (or Sau1.5 according to Repbase nomenclature), was mapped to the CEN and subTel regions of the chromosome 5 short arm and has a 33 bp monomer. The estimation conducted by fiber-FISH shows two SatCH5 TR arrays of 250–500 kb [11].

The Chinese hamster chromosome set consists of 22 chromosomes (10 pairs of autosomes and XY sex chromosomes) that differ in size and morphology. The karyotype combination of metacentrics and acrocentrics of different sizes is unusual for mouse-like rodents (Myomorpha). In the mouse (*Mus musculus*) and the Syrian hamster (*Mesocricetus auratus*), karyotypes contain many acrocentric (*M. musculus*) or metacentric (*M. auratus*) chromosomes of similar size [12,13]. It is difficult to recognize individual chromosomes by karyotyping or to reveal them by sorting [14]. The high degree of chromosome diversity in Chinese hamster karyotypes presupposes that TR homogenization between chromosomes faced severe difficulties. The appearance of chromosome-specific TR families that are not features of the mouse or Syrian hamster [9,15] could be expected as a consequence. Most of the Chinese hamster chromosomes can be identified by conventional staining, which makes them convenient for cytogenetic research.

Chinese hamster ovary cell lines (CHO) represent a large family of related, but quite different, cell lines that are industrially relevant. The unique plasticity of the CHO genome made these cells the major mammalian host cells for the manufacturing of protein pharmaceuticals. CHO is important enough to deserve its own sequencing: assembly AFTD, used in the current work as one of the TR sources arising from CHO. A detailed G-banding comparative karyotype analysis of CHO derivatives has been carried out. Subclones of CHO cell lines are characterized by multiple complex chromosome rearrangements studied by classical cytogenetic methods, but Robertsonian translocation has not been described [16,17,18,19]. Chromosome rearrangements are non-random and correspond to individual chromosome instability in CHO cell lines. Chromosome rearrangements are often associated with heterochromatic regions [18,19], so the TR set described in the current work could be the tool to trace such rearrangements.

**Figure 1:**
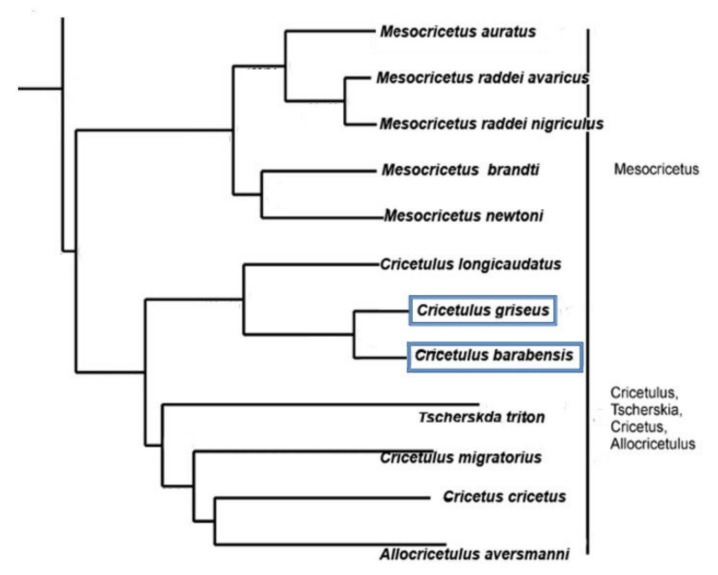
Molecular phylogeny of the *Cricetinae* subfamily based on the mitochondrial cytochrome b and 12S rRNA genes and the nuclear vWF gene ([27] adapted).

The Chinese hamster and striped hamster (*Cricetulus barabensis*) are very closely related species (Figure 1). Their karyotypes differ from each other by one Robertsonian rearrangement and X-chromosome morphology. The *C. barabensis* chromosome set is composed of 20 chromosomes [20,21]. It is proposed that striped hamster chromosome 4 arose from Robertsonian translocation of the 6 and 7 Chinese hamster acrocentric chromosomes and was apparently followed by a loss of periCEN heterochromatin [22]. The X chromosomes of Chinese and striped hamsters differ in shape—submetacentric and subtelocentric, respectively—as a result of pericentric inversion [22,23,24]. It was previously assumed that the “*griseus*” and “*barabensis*” karyotypes were derived from an ancestral karyotype 2n = 24 by two centric fusions [22,24]. More recent FISH-based ancestral reconstruction [25] and topology of the mitochondrial tree [21] support the presumption that the ancestral karyotype consists of 20 chromosomes and the *C. griseus* chromosome set is formed by chromosome fission. The fast karyotype evolution is the feature of the genus *Cricetulus* [22,26].

The level of TR sequence evolutional variability is high irrespective of whether the TR were cloned or extracted by bioinformatics from the genomes of different species [7,28]. The aim of the current work was to trace the TR distribution on chromosomes in two very closely related species. *C. barabensis* is the closest species to *C. griseus* but its genome has not yet been sequenced. Therefore, we classified the *C. griseus* TR with the assemblies available and then compared the mode of the TR distribution in *C. griseus* and *C. barabensis.* The distribution of TR probes revealed a high degree of variability even in closely related species.

## 2. Materials and Methods

### 2.1. Genome Assemblies

Chinese hamsters’ genome sequences were obtained from NCBI ftp site in FASTA format: whole genome sequencing (WGS) of *Cricetulus griseus* ovary cell culture CHO-K1 (GenBank: AFTD00000000.1) [29] (AFTD); WGS of *Cricetulus griseus* with sorting chromosome (GenBank: APMK00000000.1) [30] (APMK); WGS of *Cricetulus griseus* (GenBank: AMDS00000000.1) [31] AMDS. Genome assemblies’ characteristics are presented in Table 1.

### 2.2. Tandem Repeat Search

Tandem repeat (TR) search and analysis was performed with TRF (Tandem repeat finder, [32]). TRF version 4.09 [32] was used with the following parameters: match: 2, mismatch: 5, delta: 7, PM: 80, PI: 10, minscore: 50, maxperiod: 2000 [2]. TRF output analysis was performed with custom Python scripts. To search for large TR families, tandem repeat fields were compared using blastn software with the parameters evalue 10 × 10^−16^, dust = “no”. To eliminate any redundant entries from the TRF output, all embedded TR arrays were discarded; in case two arrays had the same sequence coordinates, a TR with a larger unit size was discarded.

Overlapping arrays were considered independent arrays. Repbase version 19.04 was used to compare TR with known repeats (transposable elements (TE)) and known *C. griseus* TR: SAU1.5 (Genbank ID AJ131828), HUCAFF170 (i.e., HC2sat) (X79296), pHC312 (TE) (X79295). To remove false-positive matches from Blast versus Repbase results, all matches that had been covered by repeats from Repbase with less than 80% were discarded.

Each pair of arrays was compared using blastn. We obtained a number of false-positive alignments due to the tandem nature of compared sequences. To remove false-positive or suspicious alignments we discarded all pair matches with a score less than 200. The remaining arrays were separated into families by Blast-defined similarity. Two TR were placed in the same family if they had a blastn match with a score greater than 200. Finally, each family was checked manually for errors.

### 2.3. TR Nomenclature and Estimation of Their Genome Abundance

There is no established nomenclature for TR; for the names for new TR families, we used a scheme proposed earlier [2]. The TR family name consists of two parts: the first indicates the abbreviation of the species name of the animal in the genome of which the TR family is found (CG—*Cricetulus griseus*), the numeral in the second part indicates the minimum monomer length (bp) among the TR family arrays, and the next letter is used to differentiate the TR families with the same monomer length. In the current paper, we used assemblies of only one species, *C. griseus*, so letters CG were omitted from TR names.

Program bowtie2 with parameter sensitive-local was used to evaluate TR amount in the raw reads. This program aligned each TR family with raw reads (*C. griseus* SRR329940, SRR329953, SRR803174, SRR803182). The percentage of reads aligned to TR was counted as % in the genome.

### 2.4. Probe Design

The arrays with maximum sequences homogeneity were selected for the probe design to capture maximum TR arrays of the same kind. Short one-chain oligonucleotide probes were designed in a self-made Python script. The oligonucleotides were then tested for possible discrepancies (the secondary structure, etc.) by program Primer3 [33]. The probes were synthesized (Beagle, St. Petersburg, Russia) as DNA oligonucleotides with both 3′ and 5′ ends labeled with biotin or Cy3. The sequences of the probes are listed in Table 2 according to the amount in the genome.

The TR for the experimental verification were selected with the following criteria: the high amount of the fields found, long fields, the lack of similarity with the known TE, TR presence in at least two assemblies, relatively low GC content.

### 2.5. Animals

Hamsters were transferred to St. Petersburg from the Joint-Use Center “Biosource collection” of Federal Scientific Center of the East Asia Terrestrial Biodiversity (Vladivostok, Russia). Chinese hamsters *C. griseus* originated from China and have been kept in Russia in a laboratory as the breeding line since the 1970s.

Three samples of striped hamsters (1 male and 2 females) were from Amur area (50°08 35 c. w. 128°14 10 in. d.). All animals were caught during the year 2017 and kept in the same center until the experiments in 2018.

### 2.6. Statement of Ethics

All applicable international, national, and/or institutional guidelines for the care and use of animals were followed. The experiments were carried out in accordance with the Animal Welfare Assurance (Assurance Identification number F18-00380) of the Institute of Cytology, Russian Academy of Sciences (valid from 12 October 2017 to 31 October 2022), for the protection of animals that are reared at experimental farms and used for scientific purposes.

### 2.7. Metaphase Chromosome Spreads

Chromosome spreads from bone marrow cells were made according to the standard method [34] with slight modifications [35]. Obtained cell suspension was dropped on slides, heated on the surface of water bath (50 °C), and air-dried. Before the FISH procedures, the slides were stored at −20 °C.

### 2.8. FISH

FISH with single-stranded oligo-probes was carried out according to the protocol [36] with several modifications. The probes were synthesized with both 3’ and 5’ ends labeled by biotin. Slides with metaphase spreads were treated in RNAse (Sigma-Aldrich, R6513, Merk KGaA, Darmstadt, Germany) stock solution (10 µg/mL) diluted 1:200 with 2xSSC for 45–60 min at 37 °C and washed 3 times for 5 min with 2xSSC at RT. Metaphase spreads were denatured in solution (70% formamide, 2xSSC) for 3–5 min at 72 °C and dehydrated in an ethanol series at −20 °C. Then, slides were incubated in the hybridization mixture: biotinylated oligo-probe in Hybrisol (Molecular Probes, Eugene, OR, USA) for 16–18 h at 37 °C. After post-hybridization washing, the slides were incubated with streptavidin conjugated with Alexa 546 (ThermoFisher Scientific, Waltham, MA, USA). Biotinylated antistreptavidin (Vector Laboratories, Burlingame, CA, USA) was used to amplify the signal, and then again streptavidin conjugated with Alexa 546 was used; all concentrations corresponded to the protocol of the manufacturer. The slides were finally mounted in Prolong Gold Antifade with DAPI (ThermoFisher Scientific, Waltham, MA, USA) and stored refrigerated in the dark.

### 2.9. Microscopy and Image Acquisition

The preparations were studied using a LEICA TCS SP5 (Leica Microsystems, Wetzlar, Germany) laser scanning confocal microscope in the Institute of Cytology, Russian Academy of Sciences, St. Petersburg, Russia. Chromosome identification was made according to the international Chinese hamster nomenclature [37]. About 20 metaphase spreads were analyzed for every TR oligo-probe and 3–5 probes were karyotyped.

## 3. Results

### 3.1. Tandem Repeats Common for Assemblies

Three assemblies were used for the TR search to minimize the potential mistakes for in silico predictions known for probable bias, and the TR families common for all three assemblies were taken into consideration (Table 3). The TR family nomenclature is given in the Section 2. In the current paper, we used the assemblies of only one species, *C. griseus*, so the letters CG were omitted from TR names.

The TR families selected for FISH verification are shown in bold. The two TR that were present in two assemblies only (62A and 27A) were selected as they possess long arrays (for example, 62A > 20 kb in AFTD) and are reasonable in content (~0.01%).

The Repbase TR SAU1.5 is determined as 33A according to the mostly short monomer and because it is well represented in the genome, ~0.5% (Table 4). It became clear why TR SAU1.5 was the 1st to be cloned: the amount of its arrays is 65% out of all the TR arrays found. The cloned HC2sat does not correspond to any of the TR found due to its composition of short simple repeats.

There are 579 fields grouped in 116 families in the AMDS assembly. The most numerous families possess similarities with TE (272A—B1 and 767A—ERV). The following TR families, 33A and 6A (~0.5% each), 79A (~0.4%) and 25 B (~0.2%), do not have any similarities with TE.

In the assembly AFTD, there are 549 TR fields split into 93 families. The two major TR families are also similar to TE - same as in AMDS 272A (B1) and 11A with similarity to the ERV2. A total of 6 out of 93 families have similarities to TE. Among the TR without similarities to TE, the main part is common for three assemblies (33A, 79A, 25B) with about half of the overall TR array amount being 33A.

The TR set comparisons among the three assemblies show some differences: 28 TR families are common for all assemblies, 26 TR families are common for only AFTD and AMDS, and 3 families are common for each of the following pairs (APMK and AFTD, APMK and AMDS). Furthermore, 60 TR families are common in at least two assemblies (Table 3).

Special attention was paid to the APMK assembly for it occurs on the sorted chromosomes and provides the possibility to determine the TR position on a certain chromosome. Again, many TR have similarities with TE or its fragments. For example, the 49A TR has similarities with ERVs fragments (Table 4).

The majority of TR families, represented by several fields in the APMK assembly, happened to show chromosome specificity in in silico prediction. They are 33A (chromosome 5), 17A (chromosome 6), 25A (chromosome 5), 62A (chromosome 2), 26A (X chromosome), etc. Moreover, the TR families with similarities to TE also have predicted chromosome specificity. Several TR families were located in silico on more than one chromosome: 79A, 6A, 77A, 18A, and some others. Several fields were found for well-represented 11A (ten fields), and in silico prediction located them to the small chromosomes 9 and 10 that could not be separated during sorting. The bulk of the 79A fields came to chromosome 5 though some fields were present on four other chromosomes (6, 9–10 and X) (Table 4).

It looks as though the hamster genome contains more TE-based TR than mouse genome [2]. The TR found in three assemblies allow us to develop reliable probes suitable for in situ verification. The TE-based TR and TR with simple sequences were sorted out.

### 3.2. FISH Mapping on Chinese and Striped Hamsters’ Chromosomes

Thirteen probes were selected for in situ mapping: 11 with maximum representation in all assemblies and 2 with arrays more than 20 kb in any of the assemblies. None of these probes has any similarity with TE. FISH was conducted with short oligo probes developed from sequences found in silico (Table 2). The obtained pattern of TR distribution indicates that TR, for which the probes were developed, really exists and the probes are quite specific.

#### 3.2.1. Chinese Hamster *C. griseus*

**Figure 2:**
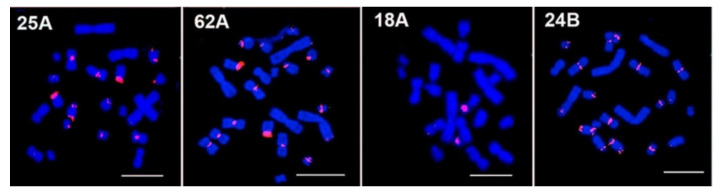
*C. griseus* metaphase plates after FISH with the TR (tandem repeat) probes. Order of the probes is according to Table 4. Those TR probes are shown that did not give any answer on *C. barabensis* plates. Bar 10 mcm.

**Figure 3:**
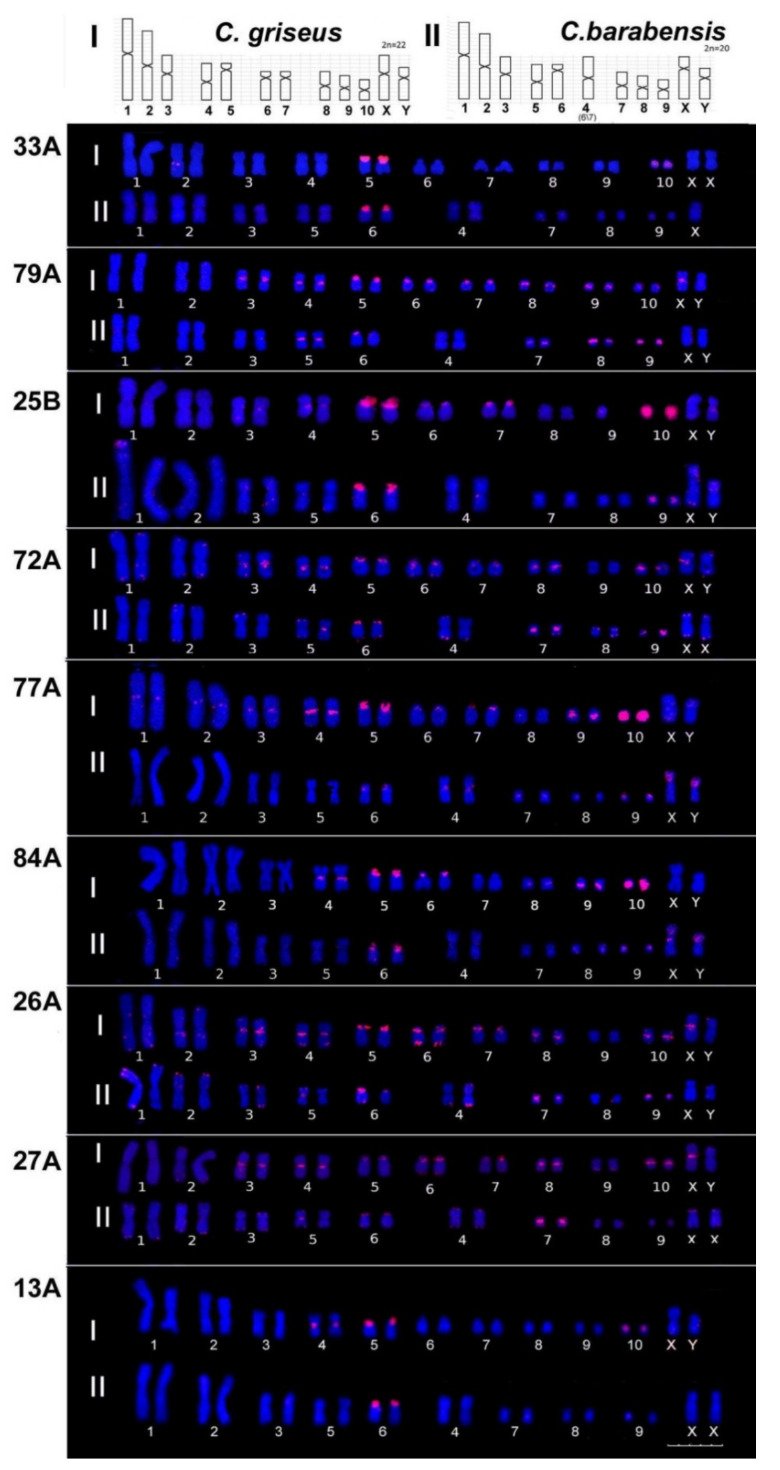
*C. griseus* (I) and *C. barabensis* (II) karyotypes after FISH with the TR probes indicated. Top row—chromosomes’ schemes and their numbers for both species. The number of chromosomes 6/7 of *C. griseus*, which corresponds to chromosome 4 of *C. barabensis*, is given in brackets. Order of the probes is arranged according to Table 2. Only the TR probes that gave the signal on *C. barabensis* methaphase plates are shown. Bar 10 mcm.

Most of the probes give a prominent signal on chromosome 5, which is known to possess a large block of C-positive heterochromatin [22,38]. For two TR (27A and 72 A), the signal on chromosome 5 is not the main one but is distributed among a number of chromosomes (Table 5 and Table 6). The major TR 33A is situated at chromosome 5, with a minor diffuse signal on chromosome 10 and sometimes on chromosome 2; this is a rare case when in silico prediction (Table 4) is almost confirmed by in situ mapping. TR 18A shows signals on chromosomes 4, 5, and 10 (Figure 2), whereas other chromosomes could be expected from in silico prediction (Table 4). For most TRs (79A, 72A, 26A, 25A, 27A, 62A, 24B) signals are located on the medium-sized or small (3–10) and also the sex chromosomes; big chromosomes (1, 2) have negligible signals in the CEN region (77A). Big chromosomes have TR in the subTel region (72A, 26A, 25A, 24B, 62A). A subTel signal was also registered on medium-sized chromosomes for several TR (Table 6). The X chromosome is known to be enriched with TR [39] but it contained only part of the TR set tested. The determined TR positions (Figure 2 and Figure 3) were summarized and compared with those of *C. barabensis* (Table 6 and Figure 3).

#### 3.2.2. Striped Hamster *C. barabensis*

The striped hamster karyotype has 20 chromosomes and differs by its rearrangement (fusion) of chromosomes 6 and 7 of the Chinese hamster and it also produces chromosome 4 (2n = 20). The decline of chromosome numbers in striped hamster karyotypes leads to a shift in their numbers—chromosome 5 of the Chinese hamster corresponds to chromosome 6 of the striped hamster (Figure 3, top row; Table 5). The pattern of TR distribution on striped hamster chromosomes differs significantly from that on Chinese hamster chromosomes (Figure 3). A total of 4 (25A, 62A, 18A, 24B) out of 13 TR tested did not give any signals on striped hamster metaphase chromosomes (Figure 2, Table 6). The striped hamster is similar to the Chinese hamster with the major signal on chromosome 6 (5 in the Chinese hamster) for the majority of the TR tested except for 77A. The TR, 77A, is an example of redistribution: in striped hamsters, the main signals belong to chromosome 9 and both sex chromosomes. Chromosome 4 (6/7 in the Chinese hamster) lacks the signal of most TR (79A, 25B, 72A, 84A, 26A, 27A) that give signals on both Chinese hamster chromosomes 6 and 7, with the exception of 77A. The signal of 79A is absent from chromosomes 4 (6/7 in the Chinese hamster), 3, and X. For 25B, an additional signal appears at the short arms of the X and Y chromosomes. The signal of 33A can be observed only on chromosome 6 (5 in the Chinese hamster). Four striped hamster chromosomes show a 72A signal instead of eight Chinese hamster ones, but additional signals appear on eight chromosome subTel regions. Similar changes could be traced with the rest of the TR probes (Table 6). Overall, the intensity of signals on striped hamster chromosomes is significantly less than on Chinese hamster ones; all rearrangements are in the heterochromatic regions.

In spite of the close kinship of the two hamster species, the TR distributions vary significantly. Changes in the TR content and position on chromosomes do not always correspond to the known chromosome rearrangement.

### 3.3. TR Heteromorphism on Homologous Chromosomes of Striped Hamsters

Homologous chromosomes’ centromeric heterochromatin block size variations may potentially reflect their population variability. This polymorphism could be checked at the level of major TRs. TR probes may serve as a reliable test to assess heterochromatin block variability. One might suppose that the heteromorphism of large centromeric heterochromatin blocks, visible in many chromosome sets, is associated with natural polymorphism within populations or cross hybridization of adult hamsters from different natural populations.

**Figure 4:**
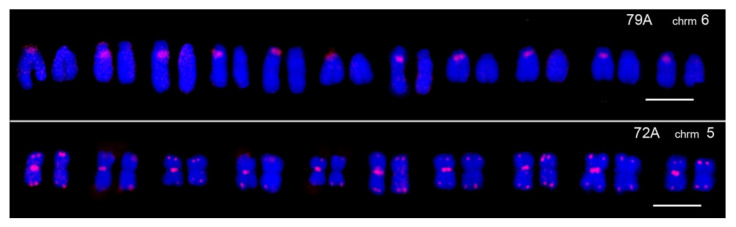
Signals’ variability on homologous chromosome 6 (upper row) and 5 (lower row) of *C. barabensis* is presented, and the probes that were used are indicated. Pairs of homologous chromosomes from different metaphase plates are shown. Bar 5 mcm.

Chromosomes 5 and 6 of *C. barabensis* give the most demonstrative examples of TR variability on homologous chromosomes (Figure 4). A similar picture may also be observed with some TR probes on other chromosomes, especially chromosome 3. It is quite common that only one homolog is labeled, whereas the other one is free of signals. The signals’ position is restricted to the periCEN chromosome regions, is known as heterochromatic, and is enriched with TR. The homologous TR heteromorphism has never been observed in *C. griseus*. Besides species-specific probes used for Chinese hamsters, this species was kept in a laboratory for years (see the Section 2), which presupposes inbreeding and the consequent loss of homologous chromosome polymorphism. Therefore, *C. griseus* is the stable species according to the absence of TR homologous polymorphism instead of *C. barabensis.* C-blocks of stable and variable constitutive heterochromatin were demonstrated in *C. barabensis* chromosomes [20]. It is noteworthy that many homologous chromosomes and their regions in these species have displayed some variation in banding patterns, thus suggesting a number of inversions in the evolution of the *Cricetus* group [22]. Heteromorphism caused by different chromosomal rearrangements is frequently observed, especially among relatively young and fast-evolving species [40]. For example, karyological data on muroid voles (genus *Microtus*) originally caught in nature, display homolog polymorphism, which disappears after sixteen months of breeding [35]. TR polymorphism as described here is compatible with this observation.

## 4. Discussion

### 4.1. Species

The precise and certain criteria of “species” are still lacking. Therefore, in species recognition, one should rely upon experts. Some authors treat “*griseus*” and “*barabensis*” as polymorphic *Cricetulus barabensis* [41,42], whereas others consider them as separate species [43,44]. The craniometric distinction of *C. barabensis s.l.* suggested that the *Cricetulus* group could be divided into several lineages (“*griseus*”, “*pseudogriseus*”, and 5 “*barabensis*”) [41]. The phylogenetic analysis of cytochrome b gene (cytb) sequences revealed that the status of the main lineages (karyomorphs) of *C. barabensis* is ambiguous. The cytb analysis confirms the existence of five lineages (“*griseus*”, “*pseudogriseus*”, and 3 “*barabensis*”) [21]. If the level of cytb divergence lay between 2% and 11%, it could indicate both intra-species and inter-species variations [45]. The level of cytb divergence between clades within *C. barabensis* (2.3–4.2%) is compatible with both the separate species and subspecies ranks [21]. Life map (http://lifemap.univ-lyon1.fr/explore.html (accessed on 15 January 2022) treats both hamsters as separate species.

Based on the experts’ opinions, we consider *C. griseus* and *C. barabensis* as separate species, though as very close ones. The Chinese hamsters, *C. griseus*, used in the current study have been bred in a laboratory since 1970, whereas the hamster specimens of *C. barabensis* were collected in the Amur region. We tried our best to use the species that were as comparable as possible.

### 4.2. Probes

The karyotypes of the muroid species were intensively investigated using classical cytogenetic methods [35,38]. Then, the stained probes technique was developed and applied to a comparative analysis of several species: mice [46], *Rattus norvegicus* [47], *C. griseus* [48], *Eothenomys proditor* [49], etc. The availability of new sets of probes allowed reciprocal chromosome staining between two *Cricetidae* species: *C. griseus* and *Mesocricerus auratus* [14], demonstrating great karyotypic differences between these two species.

The stained probes derived from the Chinese (*C. griseus*) and golden (*M. auratus*) hamsters were developed for the cross-species chromosome coloration in the following way: golden hamster chromosome suspensions underwent flow sorting and probes were generated by degenerative oligonucleotide priming (DOP)-PCR amplification of flow-sorted chromosomes [50]. A set of golden hamster stained probes consisted of nine probes (MAU for *M. auratus*) each representing one chromosome. DOP-PCR amplification is believed to be applied to many species including the plants and non-mammalian groups where interspersed repeats are not easily available for general amplification [50]. The great enthusiasm for this method was soon followed by a recognition of its limitations. At first, it was noticed in the reverse chromosome labeling technique. Although producing a relatively even signal for euchromatin, the DOP-PCR amplification often fails to label highly repetitive sequences in the acrocentric short arms, the CEN, and the heterochromatic regions. It is somewhat unpredictable whether these repetitive regions hybridize [51]. It seems likely that the DOP primer prefers to amplify transposable elements (TE) rather than tandem repeats (TR), suggesting that the nceresulting probes are depleted of TR [52]. The results of the hamsters’ karyotype comparison are still relevant with these limitations.

Three clades have been investigated by an approach with DOP probes in the *Cricetulus* group. Their affiliation to different species was confirmed. The differentiation among “*barabensis*” and “*griseus*” karyotypes is explained by one Robertsonian rearrangement and an inversion in the X-chromosome [20,22,24]. The karyotype of “*barabensis*” differs from “*griseus*” by the presence of the additional medium-sized metacentric chromosome (Figure 3; [22]). The differential chromosome staining shows that the metacentric chromosome 4 of *C. barabensis* consists of *C. griseus* chromosomes 6 and 7, whether it is chromosome fission or fusion. The topology of the mitochondrial tree is most consistent with the scenario implying the 2n = 20 (“*barabensis*”) ancestral karyotype.

In the current work, we used an approach which allowed us to trace the TR specifically, so that it would fill the gap in the DOP-PCR-based probes. Our approach, beginning with the TR’s determination by bioinformatic methods (set of TR produced from *C. griseus* genome available) and resulting in relatively short probes, does not allow us to observe large chromosome rearrangements but is suitable to shed light on the TR distribution in very closely related species. We consider all our probes as periCEN because none of the probes stained the CEN of all chromosomes as expected for mouse-like rodents true CEN, for example, mouse minor satellite [52].

### 4.3. Library Hypothesis

In cross-hybridization on remote species, the picture of the dispersed staining along chromosome arms is usual for initial periCEN satDNA probes [26,53]. Based on such a picture and supposing that single TR copies are beyond FISH resolution, the library hypothesis postulates that the particular TR sequence is presented, most probably, as a low copy number repeat with scattered distribution; then, for some reason, during evolution, this sequence changed its genomic organization, from initially interspersed to tandemly repeated [26,54].

The TR oligos used in the current work mostly concentrate in the periCEN region, and the staining along chromosome arms is negligible. It is intriguing that oligo probes are shorter than the probes of cloned satDNA. There are 4 TRs totally absent in *C. barabensis* (Figure 2, Table 6). These TR are not abundant in the genome, with an amount of no more than 0.02% (Table 4). Still, in the Chinese hamster their position is centromeric in a broad sense. The distribution of other TRs differs significantly, but all of them remain in heterochromatic regions: the periCEN or subTel position (C and T, Table 6). The intensity and number of signals from the TR decrease in *C. barabensis* (Figure 3, Table 6). Similarly, C-bands in *C. barabensis* are stained less intensely and are smaller in size than the C-bands of *C. griseus* [22]. It is possible that species-specific TR sets exist for *C. barabensis* in the same way as for the murine species, *M. musculus* and *M. caroli* [53]. Mouse major satellite of *M. musculus* (MaSat, ~14%) is substituted by sat79 and sat60 in *M. caroli* (~5% and ~11%) [55]. Both sat79 and sat60 of *M. caroli* do not produce any signal on *M. musculus* metaphase plates [53]. The artificially obtained hybrids of these species are of low viability and are sterile [56]. The direct comparison of the 2 species-specific TR sets could give the answer of precise TR distribution when the *C. barabensis* genome will be sequenced. Genome search programs are sophisticated enough to find even one TR monomer copy in raw reads assembled only to contigs [2]. Based on the pictures obtained, we can conclude that the main TR rearrangement is going on in the periCEN region not involving the extra sequences from the chromosome arms as the library hypothesis supposes [26,54]. The features of the DNA replication under replication stress [57], which is inevitable during speciation, are sufficient to cause the amplification of the TR monomer, which varied just in the periCEN region.

### 4.4. CHO (Chinese Hamster Ovary) Cell Lines

Some hints of TR rearrangement during speciation revealed in this work could be found in hamster chromosome rearrangements *in vitro*. Chinese hamster ovary cell lines (CHO) were obtained from a biopsy of the ovary of an adult female. The emergence of CHO original cells was accompanied by the partial loss of the second and X chromosomes, so the modal chromosome number is 20. One normal chromosome homologue exists in the chromosomes of 1, 5, and 9 pairs; apparently, these chromosomes represent the most stable (i.e., conserved) part of the CHO karyotype. The variable part of the CHO karyotype is represented by chromosomes X, 1 (2nd homologue), 5 (2nd homologue), 7, 8, and 10. Rearrangements of these chromosomes determine the genetic diversity and individuality of the chromosome set structure of different CHO cell lines. DNA copy variations affect predominantly the same chromosomes, which are X, 7, 9/10, as well as chromosomes 5 and 6, as shown by genome sequencing of the different CHO cell lines [58]. The breakpoints in chromosomes involved in the interchromosome rearrangements are often located in the CEN and periCEN regions [19], exactly the ones traced in the current work.

The CHO chromosome set of adherent monolayer and suspended lines has been compared and revealed a high degree of original karyotype rearrangement. A normal homolog for chromosomes 1, 2, 4, 5, and 8 was observed, but both homologs for the 3, 6, 7, and 10 chromosomes were totally rearranged and so represent the most variable part of the genome [18]. We traced the difference in TR distribution on similar chromosomes (Figure 3, Table 6). The Chinese hamster chromosome 5 contains the nucleolus organizing region [59] and probably due to this, the rearrangement was prohibited in one homolog [18,19] and coincided with conserved TR distribution between species (Figure 3).

In the current study, we can consider chromosome pairs 1 and 5 as stable chromosome homologs. Consequently, only one TR (77A) for these chromosomes shows a difference between the 2 species (Figure 3). Chromosomes 6 and 7 proved their variability, being involved in Robertsonian translocation producing chromosome 4 of *C. barabensis* with a loss of the periCEN heterochromatin [22]. This loss is visible for most of the TR from the 6 and 7 chromosomes of *C. griseus* and in chromosome 4 of *C. barabensis* (Figure 3). Chromosomes 9/10, which belong to the variable ones according to sequencing data, also show a prominent variability between the two species (Figure 3). The *C. barabensis* 5 and 6 chromosomes also show prominent TR copy number variation between the homologous chromosomes (Figure 4). So, the original hamster karyotype variations traced in vitro for CHO lines correspond to the ones observed in the current work to some extent. The set of TR tested could be used to trace the degree of stability of CHO sub-lines during the establishment of a high and stable producer. 

### 4.5. Interspecies Hybrids

In wildlife, the cross-hybridization between the species determined as *C. griseus* and *C. barabensis* is apparently absent [60]; the existence of a few potentially hybrid specimens is questionable [61]. Hybrids between these species could be obtained in the laboratory; they are viable but with reduced fertility [20,62]. Hybridization experiments suggest a lack of post-zygotic isolation [62] though meiosis was not investigated in the sense of synapsis.

Hybridization and hybrid sterility have been studied in other muroid species. In the hamster genus, *Calomyscus*, centric and tandem fusions and heterochromatin variations play a major role in the karyotype evolution [63]. The use of chromosome-specific staining supports the hypothesis of high rates of chromosomal transformations by the translocation and variation of heterochromatin [64]. Morphometric and molecular genetic analyses of the diversity show that the genus demonstrates a lack of correlation between its karyotypic and morphometric structure, as well as the reproductive incompatibility of various forms.

Different chromosomal rearrangements in different species of the gray voles apparently played a minor role in the genesis of hybrid sterility. For example, the chromosomal forms of *Microtus arvalis*, “*arvalis*”, and “*obscurus*” that differ in a series of pericentric inversions and CEN shifts [65,66,67], produce fertile male and female hybrids. Multiple chromosomes’ rearrangements, such as Robertsonian translocations and tandem translocations in the vole *Alexandromys evoronenesis* of two races, did not influence offspring wellbeing [35]. More distant species of voles reveal hybrid sterility, which has been studied [68]. Species that were examined showed intrinsic postzygotic isolation in the form of male hybrid sterility caused by chromosomes un-pairing during meiosis. In the sterile hybrids, asynapsis or delayed synapsis of the small chromosomes was observed [68]. An X–Y asynapsis in the dwarf hamster hybrids was the main cause of their sterility [69].

We would like to suppose that only species with the same TR sets can produce viable offspring if the number of chromosomes does not change dramatically (for example, by polyploidization). The TR sets of the majority of animals are not yet determined. Progress in sequencing and TR investigation makes it possible to compare species-specific sets of TR. Such a comparison could prove that the existence of similar TR sets may guarantee a successful pairing not disturbed by asynapsis. Future TR sets’ determination in different clades and kingdom groups will be able to provide additional species criteria.

## 5. Conclusions

*C. barabensis* chromosome 4, which is supposed to be a result of 6 and 7 *C. griseus* Robertsonian translocation, lost some heterochromatic blocks according to TR hybridization (Table 6). *C. griseus* and *C. barabensis* are separate species due to the species-specific TR for the Chinese hamster and prominent differences in the TR distribution in both species. The TR variation observed within homologous *C. barabensis* chromosomes is caused by a lack of inbreeding in natural populations. Viable hybrids of two hamster species could probably be obtained due to the similarities in their TR sets.

## Figures and Tables

**Table 1:** Characteristics of Chinese hamster genome assemblies.

WGS Project	Assembly Name	Sequencing Technology	Assembly Method	Total Sequence Length	Number of Contigs	Contig N50
APMK	Cgr1.0	Illumina GA Iix	ALLPATHS-LG v. 41879	2,332,774,290	319,219	11,899
AFTD CHO	CriGri_1.0	Illumina GA Iix	SOAPdenovo v. 1.05	2,399,786,748	265,787	39,361
AMDS	C_griseus_v1.0	Illumina HiSeq	SOAPdenovo v. 2.2	2,360,130,144	218,862	27,129

**Table 2:** Oligo probes used in the current work.

No.	TR	Sequence
1	33A	GTGATGTCACCTGAAGGGTCT
2	79A	CTAGTTTTCTGTATTACGTTGTATCCG
3	25B	TGTCCTTCTCTCCCCAGTGTC
4	72A	CCTCCTAAAGACATAACTGAAATCC
5	77A	CCTTGCCTTGCCTAAATGAGA
6	84A	ACTGGAGAGAAACCCTATGAATACC
7	26A	CTAGTGCTCCTGTAAGGAAGCC
8	25A	GAAGAACCAGCTAACACTAGGC
9	27A	AGGCTGGGACAATGGAGA
10	62A	CAGCACTGTGACATCAGAATAGA
11	18A	GACAGATGAGAGCTGGGTGA
12	24B	TGGTCAGGCCTATACAGAGAG
13	13A	GTGCAGAGTGAGAGTGCAGAGAG

TR—tandem repeat.

**Table 3:** Tandem repeats common for two and three assemblies.

Assembly	TR Family	Numbers of Families
AFTD + AMDS + APMK	6A, 9A, 11A, **13A**, **18A**, 18B, 19B, 20B, 23A, 24A, **24B**, **25A**, **25B**, **26A**, **33A**, 46A, 65A, **72A**, **77A**, **79A**, **84A**, 84B, 141A, 154A, 272A, 291A, 304A, 669A	28
AFTD + AMDS	17B, 21C, 21D, 22A, 23B, 26B, 27B, 30B, 31A, 31B, 32A, 32B, 33B, 36A, 51B, 58A, 60A, 63A, 72B, 94A, 100A, 104A, 170A, 180A, 450A, 1464A	26
AFTD + APMK	20A, **62A**, 616A	3
AMDS + APMK	**27A**, 87A, 146A	3
All		60

The TR families selected for FISH verification are shown in bold.

**Table 4:** Tandem Repeats in the *C. griseus* genome.

No.	TR Family	Maximum Array Length, bp	GC, %	Amount in Genome, %	Genome Assembly	Chromosome In Silico	Repbase Similarities
1	272A	5953	44	1.0711	AMDS	X	B1
2	11A	13,947	50	0.9855	AFTD	9–10	ERV2
3	49A	2075	32	0.8666	APMK	8	ERV
4	767A	7543	42	0.66232	AMDS	NA	ERV2
5	6A	36,714	59	0.5978	AFTD	5, 6, 8–10	
6	**33A**	**29,248**	**46**	**0.5119**	**AFTD**	**5**	**SAU1.5**
7	**79A**	**12,802**	**34**	**0.4047**	**AFTD**	**5, 9–10, 6, X**	
8	**25B**	**14,645**	**49**	**0.1788**	**AMDS**	**9–10**	
9	304A	4935	36	0.1365	AFTD	NA	ERV2
10	**72A**	**40,914**	**39**	**0.1284**	**AFTD**	**1**	
11	**77A**	**3456**	**40**	**0.0761**	**APMK**	**5, 2, 8**	
12	**84A**	**2885**	**39**	**0.0715**	**APMK**	**all**	**Zn-finger**
13	**26A**	**28,887**	**45**	**0.0569**	**AFTD**	**X**	
14	17A	15,866	41	0.0340	APMK	6	
15	65A	3391	39	0.0296	APMK	X	Tc1
16	**25A**	**13,526**	**43**	**0.0232**	**APMK**	**5**	
17	**27A**	**2668**	**46**	**0.0172**	**APMK**	**6**	
18	**62A**	**8036**	**36**	**0.0076**	**APMK**	**2**	
19	**18A**	**11,035**	**49**	**0.0074**	**AFTD**	**6, 2, 9–10**	
20	**24B**	**5004**	**48**	**0.0004**	**APMK**	**7**	
21	**13A**	**1769**	**48**	**0.0003**	**APMK**	**3**	

There are 15 TR with the content prevailed in the genome assemblies (1–15) shown and additional TR, which were checked in FISH (16–21) independently of their content. The columns are: No.—TR number in the table; Maximum array length—maximum field length found in the assemblies, in bp; GC—GC content in %; Amount in genome—the amount in the assembly in %; Repbase similarities—the similarity with the repetitive elements from Repbase; Genome assembly—the name of the assembly with the maximal TR content is shown irrespective of the field’s length; Chromosome in silico—chromosomes containing each TR in silico shown (NA—not applicable). TR checked by FISH shown in bold.

**Table 5:** Accordance of *C. barabensis* chromosomes to *C. griseus* chromosomes.

*C. griseus*	1	2	3	4	5	6	7	8	9	10	X/Y
*C. barabensis*	1	2	3	5	6	4		7	8	9	X/Y

**Table 6:** Summary of the TR probe mapping in *C. griseus* and *C. barabensis*.

TR	*C. griseus*	*C. barabensis*
33A	C: 5, **10**;	C: 6 (CG5)
	**I: 2**	
79A	C: **3**, 4, 5, **6**, **7**, 8, 9, 10, **X**	C: 5, 6, 7, 8, 9 (CG: 4, 5, 8, 9, 10)
25B	C: **3**, **4**, 5, **6**, **7**, **9**, 10	C: 6, 9 (CG: 5, 10);
		**I: pX, pY**
72A	C: **3**, 4, 5, **6**, **7**, 8, 10, **X**;	C: 5, 6, 7, 9 (CG: 4, 5, 8, 10);
	T: 1, 2, 6, **Y**	T: 1–**9** (CG10), **X**
77A	C: **1**, **2**, **3**, **4**, 5, 6, 7, 9, 10;	C: 6, 4, **7**, 8, 9 (CG: 5, 6/7, **8**, 9, 10);
	I: **qX**, pY	I: **pX**, pY
84A	C: **4**, 5, **6**, **7**, **9**, 10	C: 6, 9 (CG: 5, 10);
		**I: pX, pY**
26A	C: **3**, 4, 5, **6**, **7**, 8, 10, **X**;	C: 5, 6, 7, 9 (CG: 4, 5, 8, 10);
	T: 1, 2, 3, **4**, 6, **8**, **10**, X, Y	T: 1, 2, 3, 4 (CG: 6/7), X, Y
25A	**C: 3–10, X; T: 1, 2, 6, 7, X**	**No signal**
27A	C: **3**, 4, 5, **6**, **7**, 8, **10**, **X**;	C: 5, 6, 7 (CG: 4, 5, 8);
	T: 6	**T: 1–9 (CG: −10), X**
62A	**C: 3–8, 10; T: 1, 2, 3, 6, X, Y; I: 2**	**No signal**
18A	**C: 4, 5, 10**	**No signal**
24B	**C: 3–8, 10, X; T: 1, 2, 3, 4, 6, 8, X, Y**	**No signal**
13A	C: **4**, 5, **10**;	C: 6 (CG: 5)
	**I: qX, qY**	

Chromosome numbers in bold indicate the differences between species. Indication of the signal position on chromosome: I—interstitial position on chromosome arm (p—short, q—long); T—signal at subTel; C—signal at CEN. In parentheses, the chromosome numbers of *C. barabensis* are given according to *C. griseus*, i.e., 1–3 *C. barabensis* ~ 1–3 *C. griseus*; 4 *C. barabensis* ~6/7 *C. griseus*; 5, 6, 7, 8, 9 *C. barabensis* ~4, 5, 8, 9, 10 *C. griseus.* Order of the probes corresponds with Table 4.

## Data Availability

Chinese hamsters genome sequences were obtained from the NCBI site http://www.ncbi.nlm.nih.gov/genome/ (accessed on 12 April 2018). To process the results of TRF, the original program written in Python (http://github.com/DmitriiOstr/tandem-repeat-family-finder (accessed on 12 April 2018) was used.
